# CD74 Interacts with Proteins of Enterovirus D68 To Inhibit Virus Replication

**DOI:** 10.1128/spectrum.00801-23

**Published:** 2023-07-06

**Authors:** Zichun Xiang, Zhongqin Tian, Guanying Wang, Lulu Liu, Kailin Li, Wenjing Wang, Xiaobo Lei, Lili Ren, Jianwei Wang

**Affiliations:** a NHC Key Laboratory of System Biology of Pathogens, Institute of Pathogen Biology, Chinese Academy of Medical Sciences and Peking Union Medical College, Beijing, People’s Republic of China; b Key Laboratory of Respiratory Disease Pathogenomics, Chinese Academy of Medical Sciences and Peking Union Medical College, Beijing, People’s Republic of China; c Christophe Merieux Laboratory, Institute of Pathogen Biology, Chinese Academy of Medical Sciences and Peking Union Medical College, Beijing, People’s Republic of China; Shandong First Medical University

**Keywords:** CD74, EV-D68, 2B, 3C^pro^

## Abstract

Enterovirus D68 (EV-D68) is a member of the species *Enterovirus* D in the genus *Enterovirus* of the family *Picornaviridae*. As an emerging non-polio enterovirus, EV-D68 is widely spread all over the world and causes severe neurological and respiratory illnesses. Although the intrinsic restriction factors in the cell provide a frontline defense, the molecular nature of virus-host interactions remains elusive. Here, we provide evidence that the major histocompatibility complex class II chaperone, CD74, inhibits EV-D68 replication in infected cells by interacting with the second hydrophobic region of 2B protein, while EV-D68 attenuates the antiviral role of CD74 through 3C^pro^ cleavage. 3C^pro^ cleaves CD74 at Gln-125. The equilibrium between CD74 and EV-D68 3C^pro^ determines the outcome of viral infection.

**IMPORTANCE** As an emerging non-polio enterovirus, EV-D68 is widely spread all over the world and causes severe neurological and respiratory illnesses. Here, we report that CD74 inhibits viral replication in infected cells by targeting 2B protein of EV-D68, while EV-D68 attenuates the antiviral role of CD74 through 3C^pro^ cleavage. The equilibrium between CD74 and EV-D68 3C^pro^ determines the outcome of viral infection.

## INTRODUCTION

Enterovirus D68 (EV-D68) is a member of the species *Enterovirus* D in the genus *Enterovirus* of the family *Picornaviridae*. The largest outbreak of EV-D68 occurred in 2014 in the United States and raised a great public health concern for a pandemic (1, 2). From mid-August 2014 to 15 January 2015, 1,395 cases of respiratory illnesses caused by EV-D68 were reported (https://www.cdc.gov/non-polio-enterovirus/about/ev-d68.html). In addition to respiratory infection, EV-D68 infection is also associated with polio-like acute flaccid paralysis (3, 4). As an emerging non-polio enterovirus, EV-D68 has spread throughout the world on a biennial cycle since 2014 (5). EV-D68 infections cause severe neurological and respiratory illnesses (3, 4). However, there is no specific treatment currently available for people with EV-D68 infections. The intrinsic restriction factors in the cell are constitutively present and provide a frontline defense against viruses. Therefore, screening cell-intrinsic restriction factors is a promising strategy for the development of antiviral targets.

CD74 is a type II transmembrane glycoprotein which consists of short N-terminal cytosolic region, short transmembrane region, and long C-terminal luminal region (6). Human CD74 has four isoforms: p33, p35, p41, and p43 (6). p33 and p35 are translated from the CD74 p33 mRNA by using two in-phase initiation AUG codons. P41 and p43 have a 64-amino-acid insertion encoded by the alternatively spliced exon 6b (7). CD74 was first identified in immunoprecipitates of major histocompatibility complex class II (MHC-II) molecules (8) and plays a critical role in MHC-II antigen processing by facilitating class II folding in the endoplasmic reticulum (ER) and transiting through the Golgi compartment (9). CD74 is also found as a cell surface receptor for the cytokine migration inhibitory factor (MIF) (10), d-dopachrome tautomerase (MIF-2) (11) and Helicobacter pylori proteins (12). In addition to these functions, CD74 plays an important role in viral infection. CD74 prevents viral fusion by blocking cathepsin-mediated cleavage of viral glycoproteins and protects against a wide range of cathepsin-dependent viruses, such as Ebola virus and SARS-CoV-2 (13). HIV-1 Vpu protein interacts with CD74 to reduce antigen presentation and ultimately decrease the activation of T cells, which contributes to viral persistence during HIV infection (14, 15).

To explore the role of CD74 in EV-D68 infection, we performed cleavage and mutagenesis assays of CD74 and viral replication assays to determine the impact of CD74 upon EV-D68 replication and coimmunoprecipitation to investigate the interaction of CD74 with proteins of EV-D68. We show that CD74 attenuates viral replication through interactions with the 2B protein of EV-D68 and that 3C^pro^ of EV-D68 antagonizes the antiviral activity by cleaving CD74 at Gln-125. The interaction between CD74 and EV-D68 may determine the outcome of viral infection.

## RESULTS

### CD74 inhibits EV-D68 replication in 293T and THP-1 cells.

**FIG 1:**
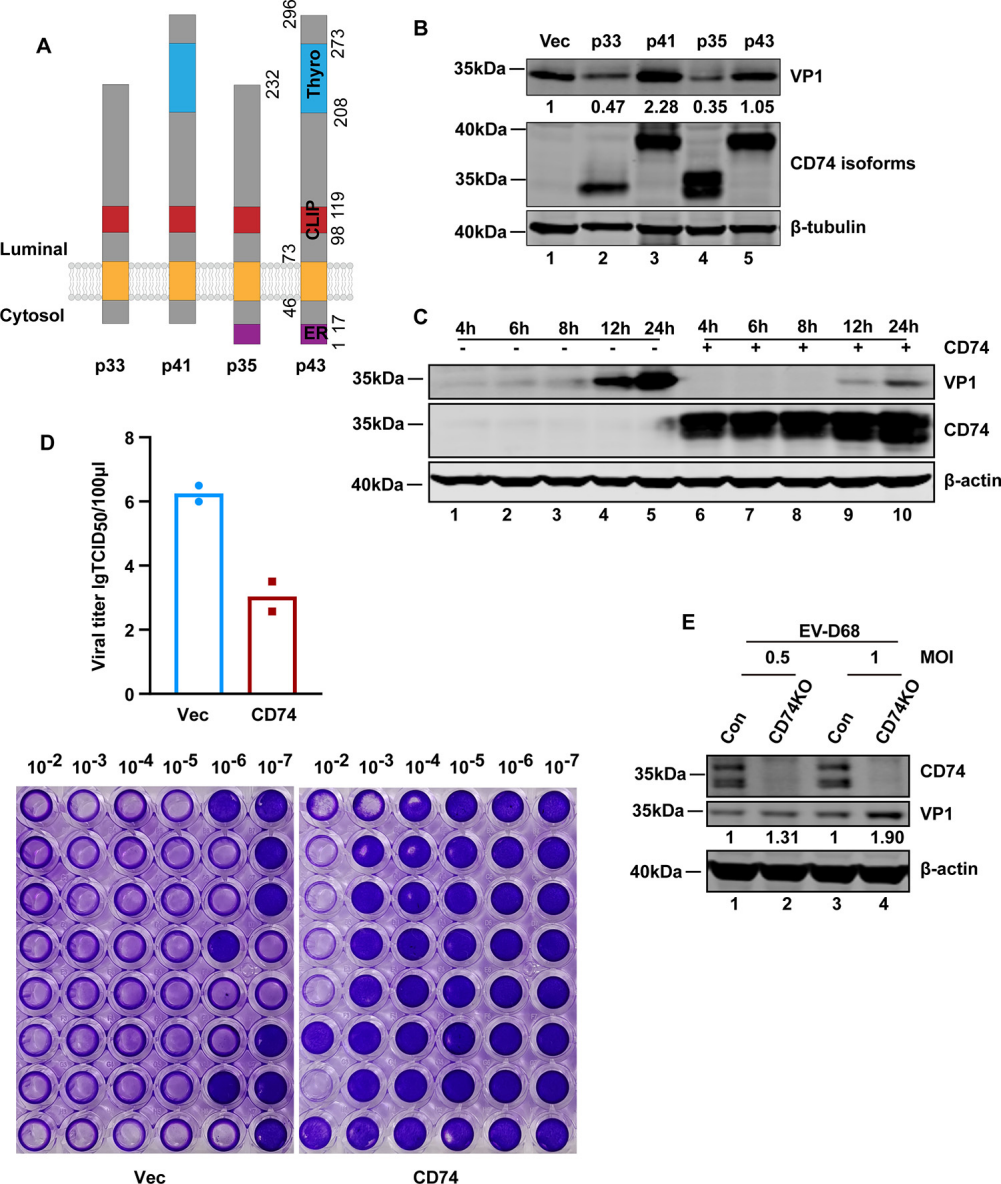
CD74 restricts EV-D68 replication. (A) Structure of the different human CD74 isoforms. The positions of the ER-retention signal (ER), transmembrane, class II-associated li chain peptide (CLIP), and thyroglobulin domains are marked. (B) 293T cells were transfected with pCMV6-p33, p41, p35, or p43 for 24 h. Cells were then mock infected or infected with a multiplicity of infection (MOI) of 0.5 of EV-D68. At 24 h after infection, cell lysates were analyzed by Western blotting with antibodies against CD74, VP1, and β-tubulin. (C) 293T cells were transfected with a control plasmid or with CD74 (p35). At 24 h after transfection, cells were infected with EV-D68 (MOI = 0.2) for 4, 6, 8, 12, and 24 h. Cell lysates were subjected to Western blot analysis with antibodies against CD74, VP1, and β-actin. (D) 293T cells were transfected with a control plasmid or with CD74 (p35). At 24 h after transfection, cells were infected with EV-D68 (MOI = 0.2) for 24 h. Supernatants were collected, and viral titers were determined in RD cells by using a TCID_50_ assay. (E) Effect of CD74^−/−^ on EV-D68 replication as determined by Western blotting. CD74 knockout THP-1 cells were infected with EV-D68 at an MOI of 0.5 or 1 for 24 h. Cell lysates were subjected to Western blot analysis with antibodies against CD74, VP1, and β-actin.

CD74 has four main isoforms which are differentiated by the N-terminal ER-retention signal and an internal thyroglobulin domain (Fig. 1A). In order to evaluate whether CD74 had any impact on EV-D68 replication, 293T cells with ectopic expression of CD74 four isoforms were challenged with EV-D68. As illustrated in Fig. 1B, only p33 and p35 decreased the VP1 expression of EV-D68, suggesting that p33 and p35 could inhibit EV-D68 replication. Because p35 and p33 use two in-phase initiation AUG codons of the same mRNA and ectopic expression of p35 plasmid showed two protein bands (35 and 33 kDa), the p35 plasmid was used below. To determine the effect of CD74 on the growth kinetics of the virus, 293T cells ectopically expressing CD74 were infected with EV-D68. At different time points postinfection, cell lysates were processed for Western blot analysis. As illustrated in Fig. 1C, the expression of VP1 verified that EV-D68 replication increased over time and CD74 decreased the VP1 expression of EV-D68. Consistent with this, CD74 overexpression reduced the efficiency of virus production (Fig. 1D). Because CD74 is highly expressed in THP1 cells and EV-D68 could replicate in THP-1 cells, CD74 knockout cell line was constructed in THP-1 cells using CRISPR-Cas9 gene editing. Then, CD74KO cells were challenged with increasing doses of EV-D68. At 24 h postinfection, cell lysates were subjected to Western blot analysis. As illustrated in Fig. 1E, VP1 expression increased in CD74KO cells, which indicated that CD74 deficiency resulted in an increase of viral infection. These results indicated that CD74 inhibits EV-D68 replication in infected cells.

### CD74 interacts with 2B of EV-D68.

**FIG 2:**
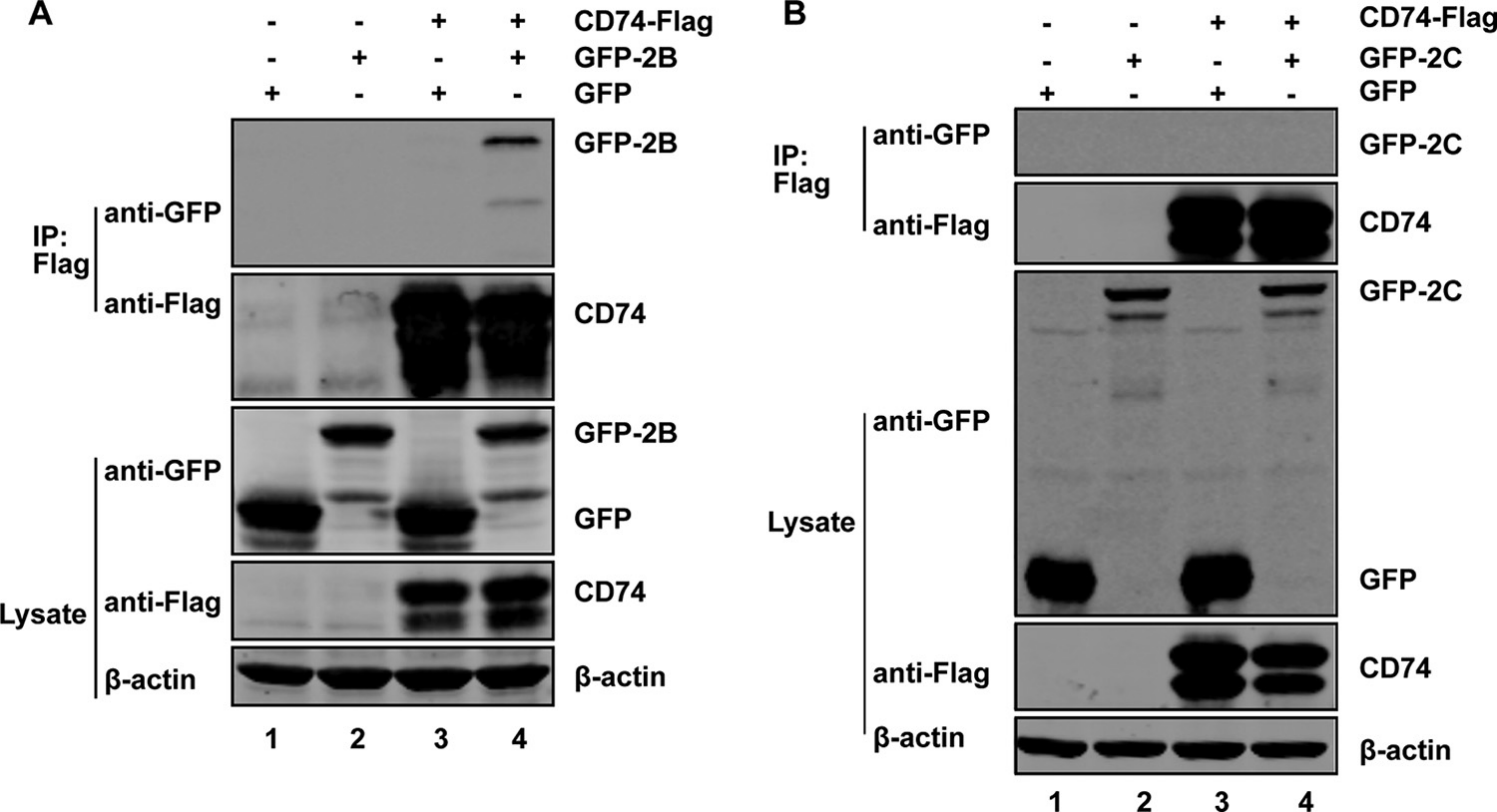
CD74 interacts with 2B of EV-D68. (A) 293T cells were transfected with CD74-Flag and GFP or GFP-2B. Cell lysates were immunoprecipitated (IP) with anti-Flag antibody. Immunoprecipitates and aliquots of cell lysates were subjected to Western blot analysis (WB) with antibodies against Flag, GFP, and β-actin. (B) 293T cells were transfected with CD74-Flag and GFP or GFP-2C. Cell lysates were immunoprecipitated (IP) with anti-Flag antibody. Immunoprecipitates and aliquots of cell lysates were subjected to Western blot analysis (WB) with antibodies against Flag, GFP, and β-actin.

2B and 2C are critical to viral replication of enteroviruses (EVs) (16–18). To explore whether CD74 interacted with 2B or 2C, 293T cells were transfected with plasmids expressing green fluorescent protein (GFP), GFP-2B or -2C and CD74-Flag. The cell lysates were immunoprecipitated with antibody against Flag tag after 24 h transfection. As shown in Fig. 2A and B, CD74 had an association with 2B but not with 2C. These results suggest that CD74 is associated with 2B in cells.

### The second hydrophobic region of 2B is responsible for interaction with CD74.

**FIG 3:**
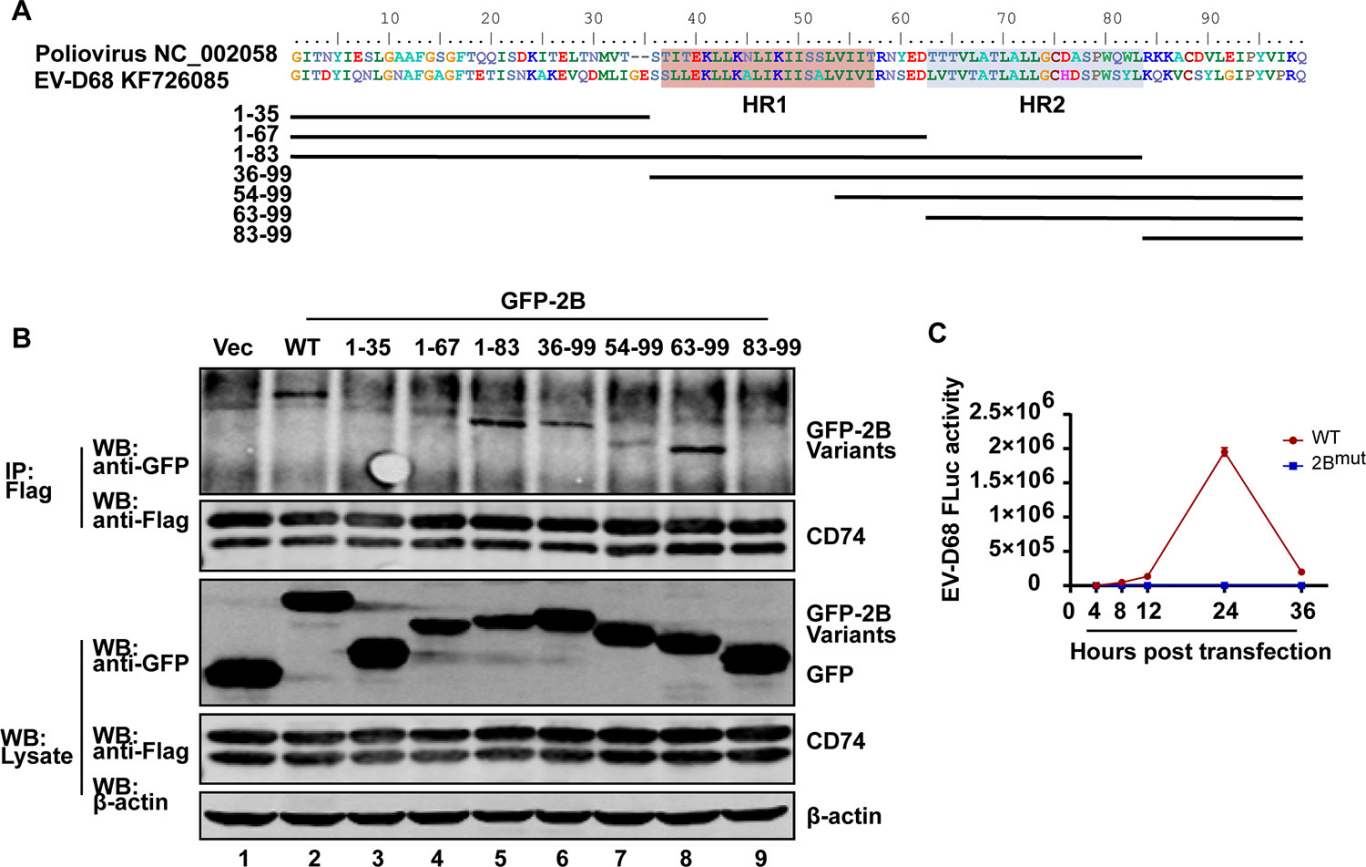
CD74 interacts with the second hydrophobic region of 2B. (A) Multiple sequence alignment of 2B in poliovirus and EV-D68 using BioEdit software version 3.3.19.0. The two hydrophobic regions are indicated. Variants of EV-D68 2B are also indicated. (B) 293T cells were transfected with CD74-Flag and GFP or GFP-2B variants. Cell lysates were immunoprecipitated (IP) with anti-Flag antibody. Immunoprecipitates and aliquots of cell lysates were subjected to Western blot analysis (WB) with antibodies against Flag, GFP, and β-actin. (C) 293T cells were transfected with RNA of EV-D68 replicons or EV-D68 2B^mut^ replicons (the second hydrophobic region of 2B deletion). Cell lysates were collected at the indicated times to assay for luciferase activities.

Similar to 2B of poliovirus, EV-D68 2B protein consists of 99 amino acid residues and has two hydrophobic transmembrane domains: HR1 (between residues 38 and 57) and HR2 (between residues 63 and 83) (19–21). As illustrated in Fig. 3A, a series of GFP-2B variants were constructed to explore the region responsible for interaction with CD74. 293T cells were transfected with plasmids expressing variants of GFP-2B and CD74-Flag. The cell lysates were immunoprecipitated with antibody against Flag tag after 24 h transfection. As shown in Fig. 3B, CD74 was associated with wild type (WT) (line 2), 1-83 (line 5), 36-99 (line 6), 54-99 (line 7), and 63-99 (line 8) fragments of GFP-2B, while fragments of GFP-2B without an HR2 region (i.e., fragments 1-35, 1-67, and 83-99) had no association with CD74. This result suggested that the second hydrophobic domain (HR2, between residues 63 and 83) of 2B was the interaction region with CD74.

To determine the critical role of HR2 domain in EV-D68 replication, EV-D68 replicon with HR2 deletion was constructed and named 2B^mut^. The RNA of EV-D68 replicon and 2B^mut^ were transfected into 293T cells, and the luciferase activity was determined at the indicated times. In contrast to the WT replicon, 2B^mut^ severely impaired the luciferase signal (Fig. 3C). These results indicate that CD74 might inhibit EV-D68 replication through interaction with the HR2 domain of 2B.

### CD74 is cleaved after EV-D68 infection.

**FIG 4:**
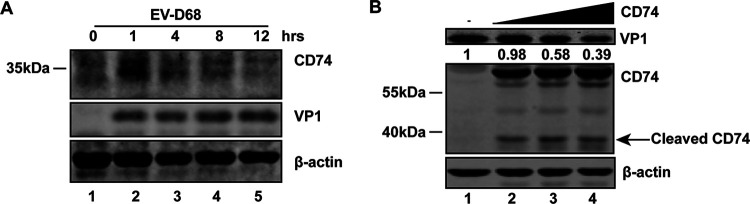
CD74 is cleaved after EV-D68 infection. (A) THP-1 cells were mock infected or infected at an MOI of 2 of EV-D68. At the indicated times after infection, cell lysates were subjected to Western blot analysis with antibodies against CD74, VP1, and β-actin. (B) 293T cells were transfected with a control plasmid or with increasing amounts of CD74 (p35). At 24 h after transfection, cells were infected with EV-D68 (MOI = 0.2) for 24 h. Cell lysates were subjected to Western blot analysis with antibodies against GFP, VP1, and β-actin. An arrow denotes 3C^pro^-induced cleavage fragments.

To determine the endogenous protein levels of CD74 in EV-D68-infected cells, THP-1 cells were mock infected or else infected with EV-D68. At the indicated times after infection, cell lysates were subjected to Western blot analysis. As illustrated in Fig. 4A, the levels of endogenous CD74 expression increased at 1 h after EV-D68 infection and decreased with the extension of infection time. This result indicated that in THP-1 cells, CD74 increased in response to EV-D68 infection but the virus decreased CD74 over time. To further confirm the effect of CD74 on EV-D68 replication, GFP-CD74 was constructed by cloning p35 into the pEGFPC1 vector, resulting in GFP fusion protein. EV-D68 infected 293T cells with ectopic expression at different doses of GFP-CD74. As indicated in Fig. 4B, ectopic expression of CD74 decreased viral replication in a dose-dependent manner. Moreover, a cleaved fragment (~40 kDa) of CD74, representing the N terminus, was detectable.

### 3C^pro^ is responsible for cleavage of CD74 in 293T cells.

**FIG 5:**
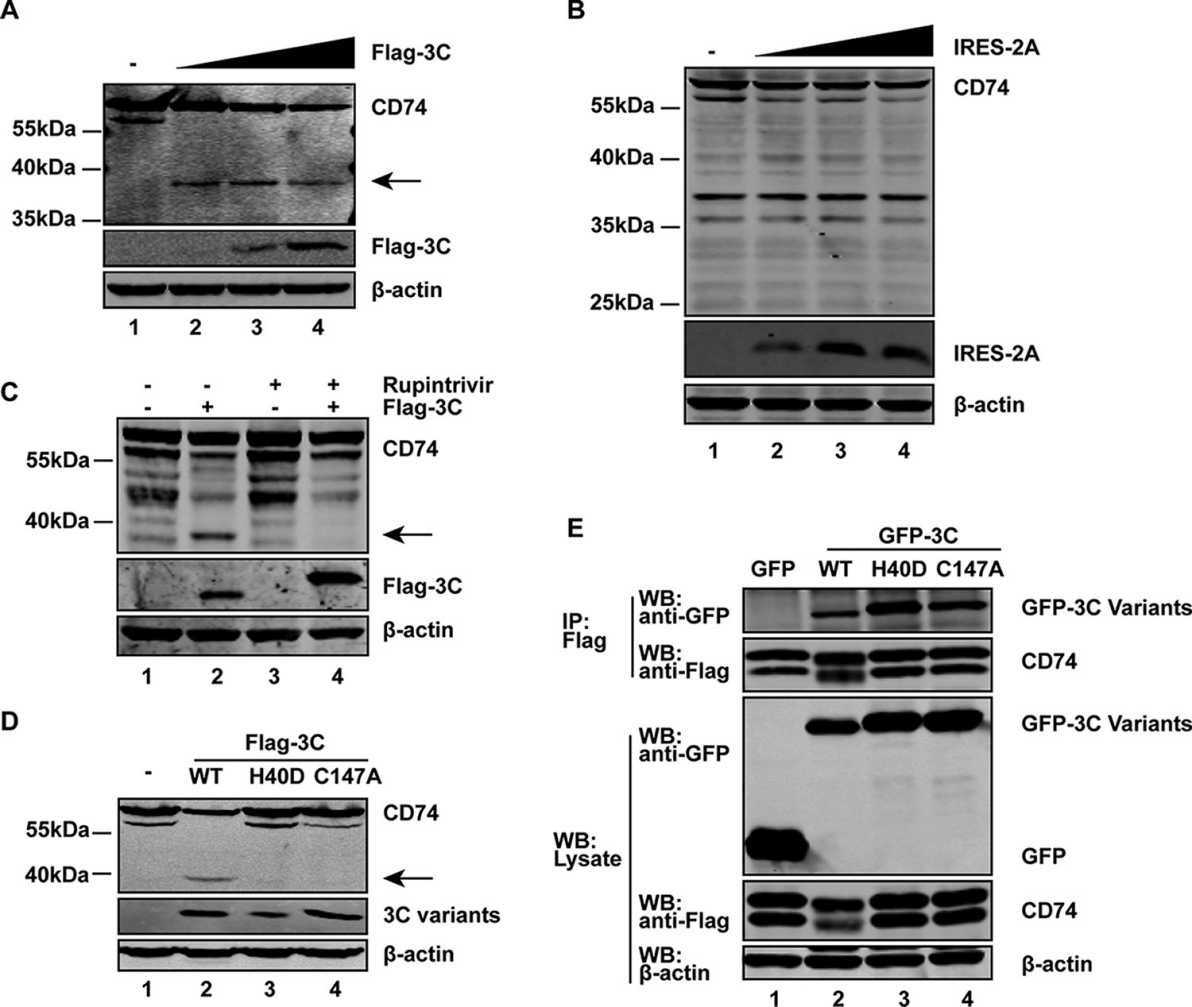
EV-D68 3C^pro^ cleaves CD74. (A) 293T cells were transfected with GFP-CD74 and a control plasmid or increasing amounts (50, 200, and 500 ng; wedge) of plasmids expressing Flag-3C. At 24 h after transfection, the cells were analyzed by Western blotting. An arrow denotes 3C^pro^-induced cleavage fragments. (B) 293T cells were transfected with CD74-Flag, along with a control plasmid or increasing amounts (50, 200, and 400 ng; wedge) of pCDNA3.1-V5-IRES-2A. At 24 h after transfection, the cells were lysed and analyzed by Western blotting with antibodies specific for Flag, V5, and β-actin. (C) Effect of the protease inhibitor rupintrivir on CD74 cleavage. 293T cells were transfected with GFP-CD74, along with control plasmids or Flag-3C. At 4 h after transfection, the cells were incubated with the protease inhibitor rupintrivir (1 μM) for 24 h. Cell lysates were then processed for Western blot analysis. An arrow denotes 3C^pro^-induced cleavage fragments. (D) 293T cells were transfected with GFP-CD74 along with control plasmids or Flag-3C variants. After 24 h, cell lysates were then processed for Western blot analysis. An arrow denotes 3C^pro^-induced cleavage fragments. (E) 293T cells were transfected with CD74-Flag and GFP or GFP-3C variants. Cell lysates were immunoprecipitated (IP) with anti-Flag antibody. Immunoprecipitates and aliquots of cell lysates were subjected to Western blot analysis (WB) with antibodies against Flag, GFP, and β-actin.

EV-D68 encodes two proteases, 2A^pro^ and 3C^pro^. To determine whether viral proteases are responsible for the cleavage of CD74 as illustrated in Fig. 4B, 293T cells were transfected with IRES-2A^pro^ or Flag-3C^pro^, along with GFP-CD74, and subjected to Western blot analysis. As indicated in Fig. 5A, 3C^pro^ cleaved CD74, producing a fragment (~40 kDa) identical to that seen with EV-D68-infected cells. On the other hand, when expressed, 2A^pro^ failed to produce the corresponding cleaved fragment of CD74 (Fig. 5B). Although there is a clear decrease in CD74 signal as the IRES-2A concentration increases, no fragments of lysis were observed. The reasons for this phenomenon might be because 2A^pro^ could halt cap-dependent mRNA translation (22). These results suggest that 3C^pro^ of EV-D68 mediates CD74 cleavage.

To determine whether its proteolytic activity is involved in CD74 cleavage. Rupintrivir, an inhibitor of 3C^pro^ with a broad spectrum of activity against picornaviruses (23), was assessed in 293T cells that transfected with GFP-CD74 and Flag-3C. As indicated in Fig. 5C, the expression of 3C^pro^ resulted in a cleaved CD74 fragment in the absence of rupintrivir (lane 2). However, when cells were treated with rupintrivir, 3C^pro^ of EV-D68 failed to mediate the cleavage of CD74 (lane 4), suggesting a role of protease activity. Since 3C^pro^ of EV-D68 bears a catalytic triad consisting of Cys147, His40, and Glu71, mutational analysis was also carried out. As shown in Fig. 5D, EV-D68 WT 3C^pro^ effectively induced the expression of a cleaved CD74 product. However, neither the H40D variant nor the C147A variant exerted CD74 cleavage, as measured by Western blotting. Immunoprecipitation assays revealed that both the wild type and these two variants associated with CD74 (Fig. 5E). These data indicate that 3C^pro^ forms a complex with CD74.

### 3C^pro^ cleaves CD74 at Gln-125.

**FIG 6:**
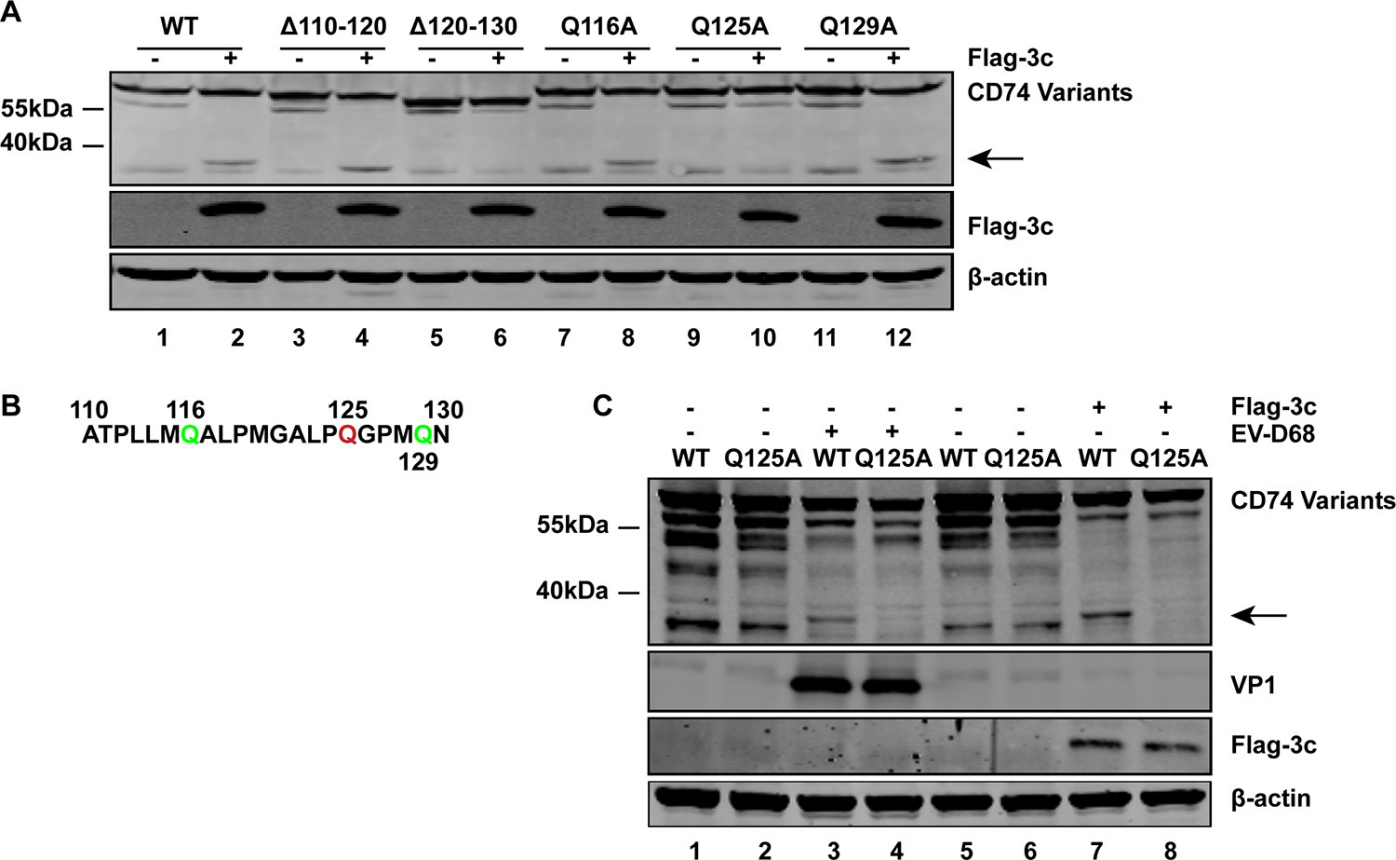
3C^pro^ cleaves CD74 at Gln-125. (A) 293T cells were transfected with plasmids encoding WT GFP-CD74 or CD74 variants as indicated, along with control plasmids (lanes 1, 3, 5, 7, 9, and 11) or Flag-3C (lanes 2, 4, 6, 8, 10, and 12). At 24 h after transfection, cell lysates were subjected to Western blot analysis with antibodies against Flag, GFP, and β-actin. An arrow denotes 3C^pro^-induced cleavage fragments. (B) Primary sequences of amino acids 110 to 130 within CD74. In this region, glutamines at sites 116, 125, and 129 were replaced with alanines. (C) 293T cells were transfected with WT GFP-CD74 or Q125A variant, along with control plasmids (lanes 1, 2, 3, 4, 5, and 6) or Flag-3C (lanes 7 and 8). At 24 h after transfection, cells were mock infected (lanes 1, 2, 5, 6, 7, and 8) or infected with EV-D68 (MOI = 0.2) (lanes 3 and 4) for 24 h. Cell lysates were subjected to Western blot analysis with antibodies against GFP, Flag, VP1, and β-actin. Arrows denote 3C^pro^-induced cleavage fragments.

Since GFP-CD74 cleavage produced one 40-kDa fragment representing the N terminus (Fig. 4B), GFP-CD74 mutants (Δ110-120 and Δ120-130) bearing an amino acid deletion from positions 110 to 120 or positions 120 to 130 were constructed. These mutants were expressed, along with Flag-3C, in 293T cells. Cell lysates were subjected to Western blot analysis. As illustrated in Fig. 6A, WT CD74 was cleaved when ectopically expressed with Flag-3C, resulting in a 40-kDa species (lane 2). Similarly, the CD74 deletion mutant Δ110-120 was cleaved in the presence of Flag-3C (lane 4). In contrast, Δ120-130 was resistant to the 3C cleavage (lane 6). Thus, the CD74 cleavage site may sit between amino acids 120 and 130. This region contains one signature sequence of 3C protease (A122XXQ125G126) (Fig. 6B). Therefore, Q125A was constructed to define the CD74 cleavage site. Q116A and Q129A were constructed as control. Q116A and Q129A remained susceptible to the 3C cleavage (Fig. 6A, lanes 8 and 12), while Q125A blocked appearance of the CD74 cleavage (lane 10). To further confirm that Q125 is the cleavage site of EV-D68, 293T cells were transfected with WT GFP-CD74 or Q125A mutant, along with 3C^pro^, or infected with EV-D68. Western blot analysis showed that WT CD74 was cleaved after EV-D68 infection or Flag-3C ectopic expression, producing the same CD74 cleavage (Fig. 6C, lanes 3 and 7). Q125A mutant blocked appearance of the CD74 cleavage both in EV-D68-infected cells and in 3C^pro^ expression cells (Fig. 6C, lanes 4 and 8). These results indicated that 3C^pro^ of EV-D68 cleaves CD74 at Gln-125.

### The cleavage fragment of CD74 by 3C is unable to inhibit EV-D68 replication.

**FIG 7:**
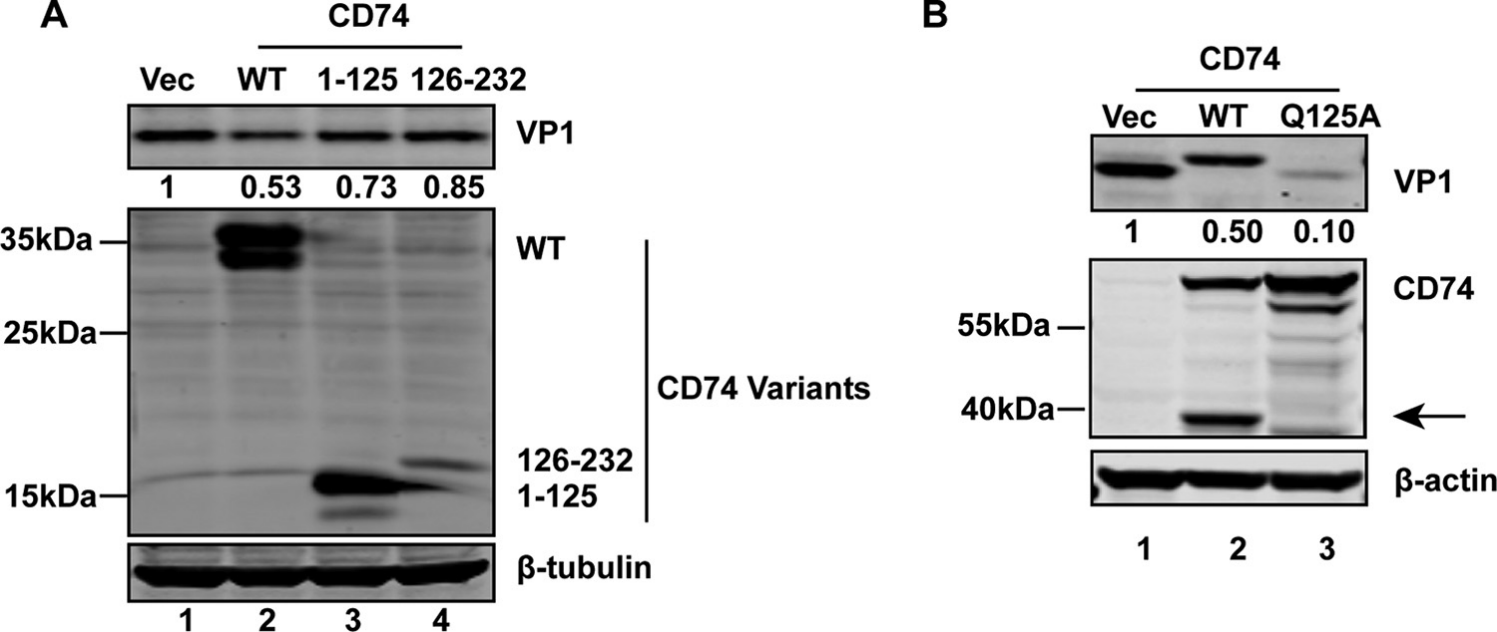
The cleavage fragment of CD74 by 3C is unable to inhibit EV-D68 replication. (A) 293T cells were transfected with WT CD74-Flag, 1-125, or 126-232 fragments. At 24 h after transfection, cells were infected with EV-D68 (MOI = 0.5) for 24 h. Cell lysates were subjected to Western blot analysis with antibodies against Flag, VP1, and β-tubulin. (B) 293T cells were transfected with increasing amounts of WT GFP-CD74 or Q125A mutant for 24 h. Cells were then infected with EV-D68 (MOI = 0.5) for 24 h. Cell lysates were subjected to Western blot analysis with antibodies against Flag, VP1, and β-actin. An arrow denotes 3C^pro^-induced cleavage fragments.

To test whether CD74 cleavage mediated by 3C^pro^ has a functional consequence, 293T cells with ectopic expression full-length and 1-125 and 126–232 fragments of CD74 were challenged with EV-D68. As illustrated in Fig. 7A, ectopic expression of full-length CD74 inhibited EV-D68 replication (lane 2), whereas the 1-125 and 126-232 fragments failed to inhibit viral replication (lanes 3 and 4). These results indicated that EV-D68 attenuate the antiviral role of CD74 through 3C^pro^ cleavage. Ectopic expression of the Q125A mutant still restricts the replication of EV-D68 (Fig. 7B).

## DISCUSSION

Enterovirus 2B is a transmembrane protein with multiple biological functions. As a viroporin, 2B involved in intracellular membrane rearrangement to form viral replication organelles (21, 24, 25). 2B also promotes virus replication and release through regulating the homeostasis of intracellular ions, cell apoptosis, and inducing autophagy (26–29). Therefore, 2B plays a vital role in enterovirus replication and antiviral treatment targeting 2B could inhibit virus replication (30, 31).

There are four isoforms—p33, p35, p41, and p43—of CD74 in humans. p33 is the prototypic and most abundant form. P33 and p35 are produced from the unique CD74 p33 mRNA by using two in-phase initiation AUG codons, while p41 and p43 result from p41 mRNA using same mechanism (7). The difference between p41 and p33 is the insertion of a thyroglobulin domain that blocks cathepsin-mediated cleavage of viral glycoproteins and protects against filoviruses and coronaviruses (13). In this study, we provide evidence that both p33 and p35, but not p41 or p43, inhibit EV-D68 replication.

Further study showed that CD74 inhibits EV-D68 replication in infected cells by targeting 2B. Coimmunoprecipitation between series variants of GFP-2B and CD74 suggested that the second hydrophobic domain of 2B was the interaction region with CD74. As two hydrophobic domains of 2B cooperated to interact with the membrane structure of the host cell (20), the interaction between CD74 and 2B could damage the role of 2B in viral replication.

Enteroviruses code two proteases, 2A^pro^ and 3C^pro^, to process the viral polyprotein. 2A^pro^ and 3C^pro^ also use viral strategies to counteract host restriction. For example, 2A^pro^ of EV-A71, coxsackievirus B3, and poliovirus cleave MDA5 and MAVS, the critical adaptors of innate immunity, to attenuate antiviral responses (32–34). EV-D68 2A^pro^ is able to cleave TRAF3 to subvert host innate immune responses (35). EV-A71 and EV-D68 3C^pro^ cleaves TRIF and IRF7 to inhibit antiviral responses (36–39). EV-A71 3C^pro^ cleaves CstF-64 to inhibit cellular polyadenylation (40). EV-A71 3C^pro^ cleaves the host zinc-finger antiviral protein (ZAP) to escape host antiviral responses (41).

In this study, we provide evidence that EV-D68 3C^pro^ cleaves CD74 at Gln-125 to antagonize the antiviral effect of CD74. CD74 overexpression inhibited EV-D68 replication, but the cleaved fragments did not show this effect. CD74 assembles into homotrimers in the ER (42). The region between amino acids 163 and 183, as well as the transmembrane section, is involved in the formation of CD74 trimers (6, 42, 43). The cleavage at Gln-125 might destroy the formation of CD74 trimers and affect its function.

In conclusion, we provide evidence that CD74 inhibits viral replication in infected cells by targeting EV-D68 2B protein while EV-D68 attenuates the antiviral role of CD74 through 3C^pro^ cleavage. The equilibrium between CD74 and EV-D68 3C^pro^ determines the outcome of viral infection.

## MATERIALS AND METHODS

### Cell lines and viruses.

293T (CRL-11268; ATCC) cells and RD (CCL-136; ATCC) cells were cultured in Dulbecco modified Eagle medium (Invitrogen, Carlsbad, CA) supplemented with 10% heat-inactivated fetal bovine serum (FBS; HyClone, Logan, UT), 100 U/mL penicillin, and 100 U/mL streptomycin at 37°C in a 5% CO_2_ humidified atmosphere. Human monocytic THP1 (TIB-202; ATCC) cells were cultured in RPMI 1640 media supplemented with 10% FBS. Low-passage cells were used after direct purchase from the ATCC, and all cells were mycoplasma-free. EV-D68 infection has been described previously (36).

### Plasmids and antibodies.

C-terminal Flag-tagged p35 and p43 of CD74 were purchased from OriGene (Rockville, MD). p33, p41, and various mutants of CD74 were constructed via PCR mutagenesis by using KOD One (Toyobo, Japan). GFP-CD74 was constructed by cloning p35 into the XhoI site of pEGFPC1 vector, resulting in GFP fusion proteins. 2B and 2C were amplified from EV-D68 stock and were cloned into the XhoI and BamHI sites of the pEGFPC1 vector, resulting in GFP fusion proteins. GFP-3C and pCDNA3.1-V5-IRESEV-D68-2A have been described previously (36, 37). Flag-3C was constructed by replacing GFP into Flag of pEGFPC1 vector. The whole genome of EV-D68 was amplified and cloned into T7 promoter downstream of pBR322 vector. EV-D68 replicon was constructed by replacing the P1 region of EV-D68 with luciferase. All variants were confirmed by subsequent sequencing.

The antibodies used in this research included CD74 antibody from Abcam (1:1,000, catalog no. ab22603), EV-D68 VP1 antibody from GeneTex (1:1,000, catalog no. GTX132313), Flag antibody from Sigma-Aldrich (1:4,000, catalog no. F3165), β-actin antibody from Sigma-Aldrich (1:4,000, catalog no. A5441), β-tubulin antibody from ZSBiO (1:1,000, catalog no. TA-10), and GFP antibody from Sigma-Aldrich (1:4,000, catalog no. G1544). A luciferase assay system was purchased from Progema (Madison, WI). IRDye 800-labeled IgG and IRDye 680-labeled IgG secondary antibodies were purchased from Li-Cor Biosciences (Lincoln, NE).

### Virus titer determination using a TCID_50_ assay.

RD cells (5 × 10^4^) were seeded in 96-well plates with growth medium (DMEM–10% FBS). The next day, 100-μL serial dilutions of EV-D68 stocks were added to the wells. The plates were then incubated for 1 h at 33°C in a CO_2_ incubator. After a washing step, the plates were incubated with minimal essential medium containing 2% FBS. The virus titers were determined using a 50% tissue culture infective dose (TCID_50_) assay after 5 days, and the cytopathic effect was observed under an inverted microscope and calculated by the Reed-Muench method.

### Generation of CRISPR-Cas9 knockout cell lines.

The CRISPR/Cas9 sequence for targeting CD74 (CACCGTGTTGGAGATAAGGTCGCGC) was cloned into lenti-CRISPRv2, which was digested by BsmBI. To construct the knockout cell lines, 1.2 μg of gRNA-expressing plasmid, 0.6 μg of VSVg plasmid, and 0.9 μg of psPAX2 vector were cotransfected into 1.2 × 10^6^ HEK293T cells. At 48 h posttransfection, the supernatants of transfected cells that contained lentivirus were collected for the following experiment.

THP-1 cells were plated into 24-well culture plate. The next day, lentivirus was added to the cells. After 48 h, cells infected by lentivirus were screened by using 1 μg/mL puromycin. Five days later, single clones were screened by a limiting-dilution cloning method. The knockout clones were verified by sequencing of the PCR fragments and Western blotting assay.

### Luciferase assays.

EV-D68 replicon or 2B mutant replicon was first linearized by MluI digestion, and then RNA was transcribed with a T7 RiboMAX Express large-scale RNA production system (Promega, Madison, WI). RNA quantity was assessed using Qubit RNA HS assay kit (Invitrogen, Carlsbad, CA). The RNA was transfected into 293T cells with DMRIE-C reagent (Invitrogen). Cells were harvested at the indicated times, and cell lysates were used to determine luciferase activities using the luciferase assay system (Promega) according to the manufacturer’s instructions.
